# Dynamic of upper body sweat distribution in young males wearing fully encapsulated chemical protective ensembles

**DOI:** 10.1038/s41598-022-04974-w

**Published:** 2022-01-19

**Authors:** Ying Chen, Chuankun Zhang, Lin Lu, Xiaohui Zheng, Suqin Chang

**Affiliations:** 1 School of Fashion, Beijing Institute of Fashion Technology, Beijing, China; 2 State Key Laboratory of NBC Protection for Civilian, Beijing, China; 3 School of Materials Design & Engineering, Beijing Institute of Fashion Technology, Beijing, China

**Keywords:** Physiology, Health occupations

## Abstract

Impermeability is a feature of fully encapsulated chemical protective ensembles (FCPE), which can affect people's sweat and affect their clothing's thermal-wet comfort. This study investigated the dynamics of upper-body sweat distribution in young males wearing FCPE and explored variations in sweat rate intra-region and inter-time for 10 young and healthy male college students. The study was carried in a climatic chamber (environment temperature 35 °C, relative humidity 60%) with participants exercising on a treadmill at 4 km/h, 5%. Sweat was collected using a 35-pad set of absorbent pads that were changed every 5 min during the course of the experiment. A total of 7-pad sets were collected with an average sweat rate of 389, 631, 920, 1137, 1100, 1211, and 1105 g m^−2^ h^−1^, respectively. The medial upper back, lateral lower back, medial upper chest, medial mid-back, and lateral top back had the highest sweat rates, with average values of 1406, 1278, 1198, 1181 and 1139 g m^−2^ h^−1^, respectively. The waist (with average values of 557, 370, 596, and 332 g m^−2^ h^−1^, respectively) and bottom zones (373, 398, 661, and 849 g m^−2^ h^−1^, respectively) had the lowest sweat rates. The above data showed that the role of FCPE in promoting body perspiration. The upper body may be split into three zones of sweat rates based on the distribution result allowing for the design of more comfortable clothing. The study includes the fundamental physiological data as well as the design recommendations for advanced personal protective equipment.

## Introduction

Chemical protective ensemble (CPE) is designed to protect workers from harmful substances, such as medical, biological, chemical, explosive, and military agents. The completely encapsulating chemical protective ensemble (FCPE) is impermeable, reducing evaporative heat dissipation and perspiration^1–4^ exposing workers to heat-related disorders. For FCPE, protection and comfort performance are few indispensable factors, and sweating is closely related to the thermal-wet comfort of the cloths.

Sweat is a key factor in garment comfort studies with^5^ regional^6,7^, age-related^8^, climate-linked gender-difference^9,10^, heat acclimation^11^, and physical level^10,12^ variations in sweating have all been studied before. These studies consistently found that the largest sweat rate (SR) was observed for the torso over the body apart from the head, whereas the extremities showed the lowest rate. Moreover, sweat rates in children and the elderly were lower than the young people. Likewise, the sweat rate in females was lower than in males^13,14^. Tropical natives originating from the long-term heat acclimation were observed to have lower body sweat loss and sweat rate than temperate natives^9,10,14,15^. However, people with short-term heat acclimation were observed to enhance sweating rates^11,13^. Sweat may be affected by the low air permeability of CPE and creates an intolerable environment involving non-steady-state heat stress exposures, which affects the steady-state sweating^2,4,16^. Ding’s study^17^ showed that there was a significant difference among the different protective levels. Thus, wearing full protective ensembles, participants sweated about 900 g for an hour. The dynamic conditions of sweat changes and sweat distribution data were deemed critical for thermal-wet comfort. Despite this, little study has been done on the sweat rate while wearing CPE.

The technical absorbent and ventilated capsule methods are the most commonly used routes to measure regional sweat rate (RSR)^5^. The technical absorbent method, allowing the simultaneous measurement of large amounts of sites across the body, is appropriate for a short, predefined period. In the case of ventilated capsule approach, most studies only measure a few sweat areas using a limited skin surface (typically 2–5 cm^2^, but up to 20 cm^2^), which is then generalized for the entire body leading to an inaccurate picture of regional relationships between these small sweat regions and the whole regions with installed ventilated capsules^3,5,18^.

This study uses a technical absorbent method to explore the sweating distribution profile for the upper body within the FCPE microclimate. Chemical protective ensemble is impermeable and creates a microenvironment isolated from the outer environment. The evaporation rate of the trunk is very low. The patches covered on the trunk affect the sweat evaporation of the human body into the clothing microenvironment, but this effect is relatively small due to its low evaporation. In places with strict requirements, it is necessary to consider the effect of the patch on sweat evaporation. In this study, the effects of regional sweating rates on the upper body are investigated along with variations in regional sweating rates from the participants exercising on a treadmill with sweat collected every 5 min for the duration of the experiment.

## Materials and methods

### Participants

Ten healthy, young male college students were recruited. Their age, height, weight, and body surface area are 23.1 ± 0.8 years, 1.77 ± 0.04 m, 65.8 ± 6.2 kg, and 1.81 ± 0.10 m^2^, respectively (mean ± standard deviation). Body surface area was calculated using the Dubois formula^19^. Participants were requested to refrain from drinking alcohol, taking coffee, taking medicines, sleeping late, and engaging in strenuous physical activity. All experimental procedures were proved by the State Key Laboratory of NBC protection for civilian ethics committee with the full consent of participants. The procedures were carried using relevant guidelines and Governmental regulations governing human research.

### Sweat pad preparation and application

**Figure 1:**
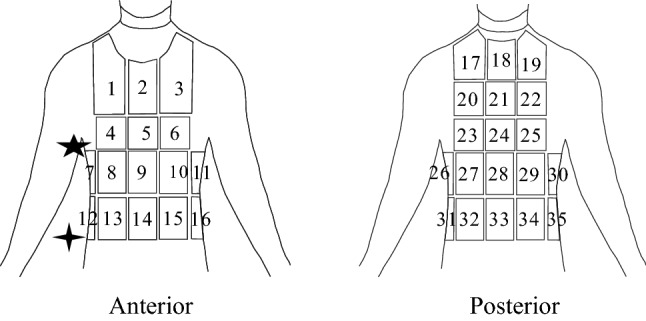
Schematic representation of the installed absorbent pads onto the upper body divided into 35 regions for profiling sweat distribution. The figure was created using Coreldraw Version (21.0.0.593).

Figure 1 shows that the absorbent pads front and back were divided into 16 and 19 areas based on the bust line, waist line, and Sacroiliac line, respectively. Table 1 shows the numbers and locations of 35 sweat pads for the upper body. During the experiment, every 5 min, a fresh set of absorbent pads was replaced. All of the absorbent pads connected to the plastic sheet had an impermeable backing to prevent sweat from evaporating. During collection, a width of 0.5–1.0 cm of absorbent pads was kept apart to separate the areas and avoid perspiration movement and contamination of neighbouring pads. The regions numbered 7, 11, 12, 16, 26, 30, 31and 35 belong to the waist region whereas 12–16 and 31–35 belong to bottom regions and 17–25, 27–29 belong to back regions.

**Table 1:** Numbers and locations of 35 sweat pads for the upper body.

Number	Location	Number	Location
1	Left upper chest	17	Left top back
2	Medial upper chest	18	Medial top back
3	Right upper chest	19	Right top back
4	Left lateral chest	20	Left upper back
5	Medial chest	21	Medial upper back
6	Right lateral chest	22	Right upper back
	★ Midaxillary		
7	Anterior left upper waist	23	Left mid back
8	Left abdomen	24	Medial mid back
9	Medial abdomen	25	Right mid back
10	Right abdomen	26	Posterior left upper waist
11	Anterior right upper waist	27	Left lower back

12	Anterior left lower waist	28	Medial lower back
13	Left lower abdomen	29	Right lower back
14	Medial lower abdomen	30	Posterior right upper waist
15	Right lower abdomen	31	Posterior left lower waist
16	Anterior right lower waist	32	Left bottom back
		33	Medial bottom back
		34	Right bottom back
		35	Posterior right lower waist

### Experimental ensembles

**Figure 2:**
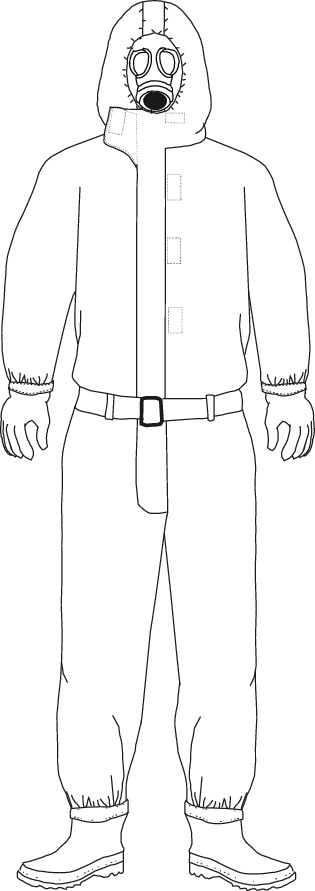
Experimental ensembles style figure^22^. The figure was created using Coreldraw Version (21.0.0.593).

FCPE ensemble included chemical protective clothing, sportswear, a mask and impermeable rubber gloves (Fig. 2). The total mass of the ensemble was 5.325 kg, with a determined thermal resistance of 1.71 clo and moisture resistance of 727.4 (m^2^ pa W^−1^) according to ASTM F12919(2005)^20^ and ASTM F2370(2005)^21^, respectively.

### Experimental protocol

**Figure 3:**
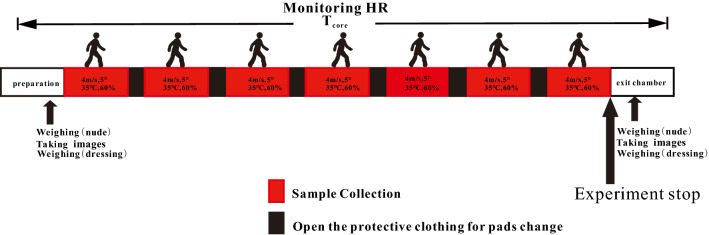
Schematic drawing of the trial protocol. The figure was created using Coreldraw Version (21.0.0.593).

Participants were given water half an hour before the trial to ensure adequately hydrated during the experiment. Each participant rested in a calm position until their pulse rate dropped, then donned experimental outfits. Participants were asked to put on a stretchy, high permeability and thin T-shirt to ensure the absorbent pads were intimately touching the skin with low, uniform pressure. The additional pads were placed over the neck region to collect the sweat running from the head and neck, which were not used in RSR measurement. Skin temperature sensors (YSI409b,4000A, YSI Inc., Dayton, America) were attached to the left body part of the participants at three locations: chest, leg and forearm with medical tape to measure skin temperature at the interval of 5 min. In a typical procedure, all-set participants were asked to exercise on the treadmill (speed 4.0 km/h, incline 5%) in the climatic chamber (35 °C, 60% relative humidity) for 5 min. The absorbent pads were refreshed every 5 min after a continuous workout. The trial protocol timeline is shown in Fig. 3.

The experimental session was terminated when one or more of the following criteria were reached: (1) participants felt unbearable pain or were physically exhausted and unable to continue; (2) core temperature reached 39.0 °C; (3) heart rate reached 180 bpm. The participants were patted before removing their absorbent pads (produced during the previous 5-min sampling period) and then stored in airtight bags, labelling and reweighing. Core temperature (${\text{T}}_{{{\text{core}}}}$) and heart rate (HR) were continuously monitored throughout the experiment. $T_{core}$ was measured by swallowing an ingestible capsule pill (HQ Inc., Palmetto, FL) where the corrected and activated pill was given 3 h before the trial. The HR was monitored continuously using a chest strap (Polar Team 2, Finland). T_*core*_ and HR were recorded at 5 min intervals before and after the experiment, the dressing weight and semi-nude weight (with shorts) were repeatedly measured using a weighing scale (type: KCC150, accuracy: ± 1 g, METTLER-TOLEDO, Zurich, Switzerland).

### Physiological parameters calculation

**Figure 4:**
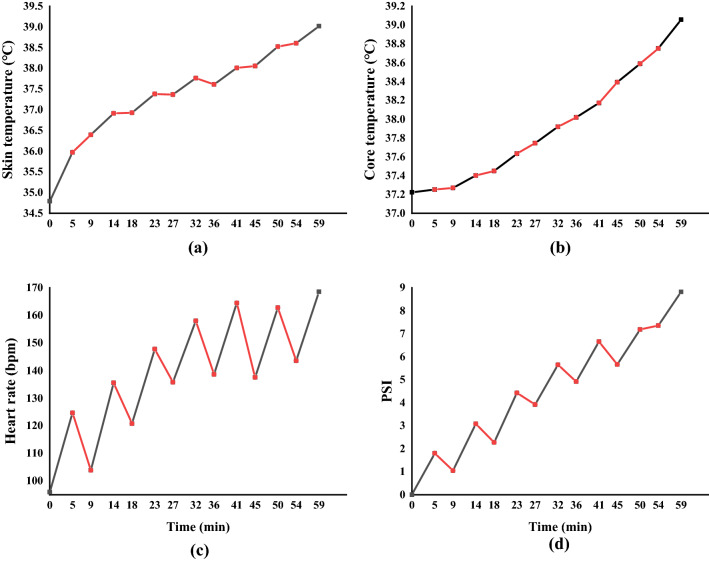
Time course physiological parameters ($\overline{T}$_sk_, $T_{core}$, HR*,* PSI) within FCPE microclimate. Black lines represent sweat collection periods. Red lines represent change pads periods. The figure was created using OriginPro Version(2018C SR1 b9.5.1.195).

Time course physiological parameters ($\overline{T}$_sk_*,* $T_{core}$*,* HR, PSI) is shown in Fig. 4. The mean skin temperature $(\overline{T}$_sk_) was calculated using Burton Eq. (1)^23^

1 $$ \begin{array}{*{20}c} {\overline{T}_{sk} = 0.50T_{chest} + 0.34T_{calf} + 0.16T_{arm} } \\ \end{array} $$

where $\overline{T}_{sk}$, $T_{chest} ,T_{calf}$, $and{ }T_{arm}$ represents mean skin temperature, chest temperature, calf temperature and arm temperature, respectively, °C.

Physiological strain index (*PSI*) was calculated using Eq. (2)^24^

2 $$ \begin{array}{*{20}c} {PSI = \frac{{5\left( {HR_{t} - HR_{0} } \right)}}{{180 - HR_{0} }} + \frac{{5\left( {T_{coret} - T_{core0} } \right)}}{{39.5 - T_{core0} }}} \\ \end{array} $$

where $$T_{coret}$$ and HR_t_ were measured during heat exposure.$$ T_{core0}$$ and HR_0_ represented the initial values, with a maximum value of 39.5 and 180, respectively.

### RSR calculation and normalized

Absorbent pads were weighed inside individually labelled airtight bags (*W*_d;_ g) before trial. The patches from all sampling periods were quickly returned to their sealed bags and reweighed (*W*_W_; g) at the end of trial. The surface area of each pad was calculated from the dry weight of each pad and the weight per unit of the surface area of the material. RSR was calculated in grams per meter square of body surface area per hour (g m^−2 ^h^−1^) using the weight change of the pad, the pad surface area (S; m^2^), and the length of time (T; min) the pad was applied to the skin. As shown in Eq. (3)^12,15^:

3 $$ \begin{array}{*{20}c} {RSR = \frac{{60*\left( {{\text{Ww}} - {\text{Wd}}} \right)}}{T*S}} \\ \end{array} $$

To standardize, individual RSR was normalized for the area-weighted sweating rate of all zones in the same period to Smith and Havenith^10^, as shown in Eq. (4). Regardless of absolute RSR data, this helps us to acquire a clearer picture of "high" and "low" and relative variations in upper body sweat zones.

4 $$ \begin{array}{*{20}c} {RSR _{norm,I} = \frac{{RSR_{i} }}{{\frac{{\mathop \sum \nolimits_{j = 1}^{j = n} \left( {area_{j} *RSR_{j} } \right)}}{{\mathop \sum \nolimits_{j = 1}^{j = n} \left( {area_{j} } \right)}}}} } \\ \end{array} $$

where $$ RSR_{i}$$ represents sweat rate in zone i in g m^−2^ h^−1^; n total number of tested zones; $$ RSR_{j}$$ regional sweat rate of zone j in g m^−2 ^h^−1^;$$ area _{j}$$ surface area of zone j.

### Upper body sweat loss calculation

Upper body sweat rate (USR) was calculated in grams per meter square of upper body surface area per hour (g m^−2 ^h^−1^) using the weight change of all pads, the pad surface area (S; m^2^), and the length of time (T; min) the pad was applied to the skin. As shown in Eq. (5).

5 $$ \begin{array}{*{20}c} {USL _{norm,I} = \frac{{USL_{i} }}{{USL_{max} }} } \\ \end{array} $$

where $$ USL_{i}$$ represented upper body sweat loss in piece measured in g; $$USL_{max}$$ was the maximum upper body sweat loss.

## Results

### Physiological responses

Physiological parameters were presented in mean $$ \pm $$ standard deviation (SD). FCPE can exacerbate physiological stress on the wearer^25^. Here, $$\overline{T}$$_*sk*_ increased from 34.$$79 \pm$$ 0.95 to 39.02 $$\pm$$ 0.36 °C (4.2 °C increase) (Fia).$$ T_{core}$$ increased from 37.22 $$\pm 0.96$$ to 39.1 $$\pm 0.33 $$ °C (1.9 °C increase) (Fig. 4b)). HR increased from 96 $$\pm 12.75 $$ bpm to 169 $$ \pm 4.50 $$ bpm (73 bpm increase) (Fig. 4c) and PSI increased from 1.8 $$\pm 0.53$$ to 8.8 $$\pm 0.54$$ (7 increase) (Fig. 4d) during measurement. Whereas $$\overline{T}$$_*sk*_ increases quickly according to the environment change during the initial stage with $$ T_{core}$$ rose faster during 5–35 min compared to 0–5 min of the trial, this was linked to environmental changes to which surface skin temperature responds quickly but core temperature takes longer to adjust^26^, and the core temperature rises faster during a workout^27^.

During the wet absorbent pads change period, $$\overline{T}$$_sk_ was almost holding steady, and $$T_{core}$$ kept rising slowly regardless of the short interruption, with a great difference between HR and PSI indicating influence of core and mean skin temperature onto sweat rate^28,29^. In this study,$$ T_{core}$$ and $$\overline{T}$$_sk_ (driver of sweat) were not significantly impacted by changing absorbent pads, hence the effect of the brief interruption to replace absorbent pads was thought to be minor and would not affect regional comparisons.

### RSR

**Figure 5:**
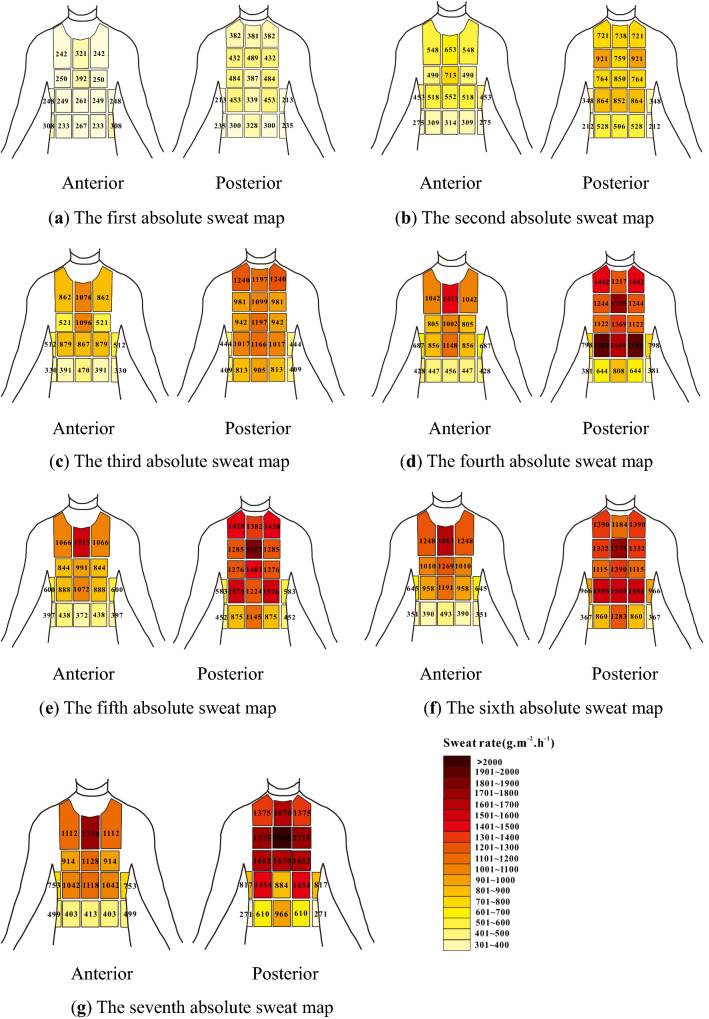
Absolute RSR of participants wearing FCPE. Figures were created using Coreldraw Version (21.0.0.593). (**a**) The first absolute sweat map was drawn based on sweat of upper body collected for 0–5 min. (**b**) The second absolute sweat map was drawn based on sweat of upper body collected for 9-14 min. (**c**) The third absolute sweat map was drawn based on sweat of upper body collected for 18–23 min. (**d**) The fourth absolute sweat map was drawn based on sweat of upper body collected for 27–32 min. (**e**) The fifth absolute sweat map was drawn based on sweat of upper body collected for 36-41 min. (**f**) The sixth absolute sweat map was drawn based on sweat of upper body collected for 45–50 min. (**g**) The seventh absolute sweat map was drawn based on sweat of upper body collected for 54–59 min. Figure 6 Relative regional sweat rates of participants wearing FCPE.

**Figure 6:**
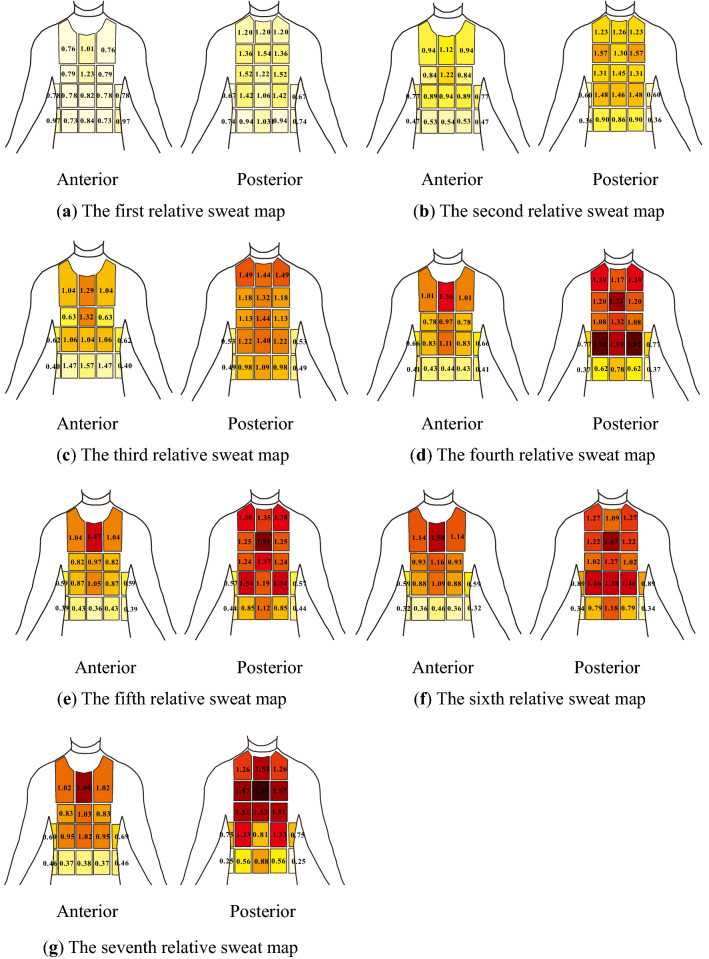
Relative regional sweat rates of participants wearing FCPE. Figures were created using Coreldraw Version (21.0.0.593).

For presenting symmetry, it was chosen to arrange comparable right-left pads to form 22 sections for study. Medians were used for graphical presentations in this study. A set of absorbent pads allowed the mapping of an upper-body sweat (Fig. 2). As shown in Fig. 5, seven upper body sweat maps of sweat distribution were created, with numbers rounded to the closest integers. RSR normalized data were showed in Fig. 6. Figure 6 shows that a value of 1 corresponds to the average sweat rate of the upper body, whereas values less than 1 and more than 1 correspond to sweat rates below and above the average sweat rate of the upper body, respectively.

## Discussion

### Dynamic upper body sweat loss

USR of seven pieces were recorded in values of 389, 631, 920, 1137, 1100, 1211 and 1105 g m^−2 ^h^−1^, respectively. Individual’s upper body sweat loss (USL) for the piece was normalized for the maximum of individual’s USL to standardize USL data. Average was used for analysis. Normalized upper body sweat loss data of seven pieces were 0.34, 0.52, 0.75, 0.92, 0.90, 0.92 and 0.90, respectively. Upper body sweat loss increased steadily from the first to fourth period but remained reasonably steady from the fourth to seventh period, with weight loss calculated for nude body weight before and after the trial was 644.5 g. Sweat loss from the upper body accounted for 29% of the weight reduction. Sweat glands in the upper body became increasingly active between the first and fourth period. Humans, on the other hand, have a restricted ability to remove heat through sweat evaporation.

### Dynamic RSR

The findings showed that as the exercise progresses, absolute RSR typically rises during the first four periods of the study. Significance levels of comparison of pre-versus post-5 min absolute data were presented with Bonferroni correction in Table 2. In terms of pre- versus post-5 min absolute RSR comparison, there was a significant increase in sweating rate in 14 individual zones (*p* <  = 0.05, including 9 zones (*P* <  = 0.01) and 4 zones (*P* <  = 0.001)) at the first four periods of the trial. After Bonferroni corrections, 5 individual zones show significantly increase (*P* <  = 0.05, including 2 zones (*P* <  = 0.001)). Most regions had a significant SR increase at the first four periods of the trial. A few regions had a significant SR increase at the fourth to seventh period. Regions were categorized into two types based on RSR alteration, one is keeping a steady-state, and the other is increasing significantly. The former includes the medial chest, medial abdomen and medial back, and the remaining parts belong to the latter.

**Table 2:** A one-way measure ANOVA was performed to analyze regional sweat rate differences before and after sweat profile presented using Bonferroni correction for post hoc comparisons.

	First–second	Second–third	Third–fourth	Fourth–fifth	Fifth–sixth	Sixth–seventh	First–seventh
Upper chest	**	**					***###
Medial upper chest							***###
Lateral chest	**		***#				***###
Medial chest	*	**			*		***###
Side1,2	**		**				***###
Lateral abdomen	***#	***###					***###
Medial abdomen							***##
Side3,4			**#			***#	***###
Lateral lower abdomen	*	*					***###
Medial lower abdomen		**					**#
Lateral top back	*	***#					***###
Medial top back		*					***###
Lateral upper back	**						***###
Medial upper back							***##
Lateral mid back						*	***###
Medial mid back							**#
Side5,6			**		**		***###
Lateral lower back	*		***###	*			***###
Medial lower back							*#
Side7,8							
Lateral bottom back	*	**	*				***##
Medial bottom back							***##

The significance level was accepted at *P* < 0.05. we performed statistical analysis using IBM SPSS (https://www.ibm.com/cn-zh/analytics/spss-statistics-software). Significance levels of comparison of pre- versus post-set absolute regional sweat rates values. Significance level for uncorrected data: *P <  = 0.05; **p <  = 0.01, ***p <  = 0.001; Significance level following Bonferroni correction: #*P* <  = 0.05; ##*p* <  = 0.01; ###*p* <  = 0.001.

### RSR

It was observed from Figs. 5 and 6 that the SR of the posterior part was greater than that of the anterior part. Comparisons between anterior zones revealed that the medial upper chest had the highest sweat rate. Lower back SR was higher in the medial and upper back regions, and some prior research found the greatest RSR in the central upper back or the central lower back^10,12^. Furthermore, the lower SR was seen in the waist and bottom areas. Furthermore, studies^10,11^ found a medial to a lateral reduction across the anterior upper body. The lateral areas SR of the posterior half, on the other hand, were bigger than the central regions SR, which may be explained by the varied experimental methodology, sweat sample area, and concave spine induced by sweating collection mistake. Sweating rates of the third sweat map where HR was 121–148 bpm compared to Smith and Havenith s’ study where target HR was 125–135 bpm for intensity 1. Sweating rates of the seventh sweat map where HR was 144–169 bpm compared to Smith and Havenith s’ study where target HR was 150–160 bpm for intensity 2. Results showed that upper body sweat rates under ECPE were greater than Smith and Havenith s’ study.

Normalized regional sweating ratio data for individual zones showed no clear shift of sweating rate distribution to the upper body. As shown in All experimental procedures were proved by the State Key Laboratory of NBC protection for civilian ethics committee with the full consent of participants. The procedures were carried using relevant guidelines and Governmental regulations governing human research.

, it was observed that the medial upper back (average 1.68) showed the highest RSR, followed by lateral lower back (average 1.56), medial mid-back (average 1.46), medial upper back (average 1.41), lateral upper back (average 1.41) and medial upper chest (average 1.41). A series of sweat maps of sweat distribution exhibited similarities, indicating (1) greater sweat rate was observed on the anterior compared to the posterior, (2) greater sweat rates on the medial upper chest and back, (3) lower sweat rates on the waist and bottom regions.

### Application

**Figure 7:**
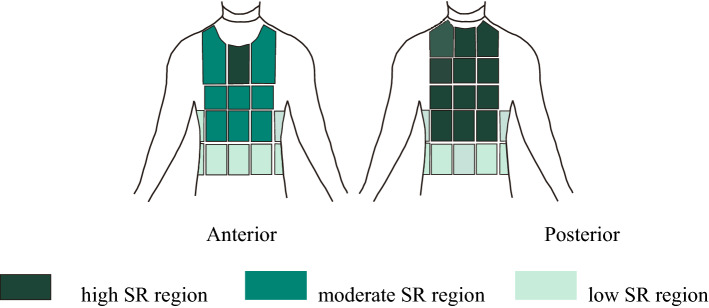
Zone map for upper body sweat rates The figure was created using Coreldraw Version (21.0.0.593).

Based on RSR alteration (Table 2) and the magnitude of RSR (Table 3) during exercise, upper body wearing FCPE can be divided into higher sweat rate zones, moderate sweat rate zones, and lower sweat rate zones, as shown in Fig. 7. The medial upper chest and back are divided into the higher SR zones, which belong to the first RSR alteration type and have a higher sweat rate, evenly sweated throughout the exercise**.** The waist and bottom regions are divided into lower SR zones, which belong to the second RSR alteration type, with a lower sweat rate. During less exercise, the remaining parts are divided into moderate SR zones, which belong to the second RSR alteration type and have moderate RSR. These regions show differences in thermal comfort exposure to cold and heat^30,31^. Abdominal cooling has a minor effect on the upper back, but it can significantly enhance temperature sensation in the lower back. For creating thermal comfort, the neck, like the face, showed more sensitivity than other parts. To maximize the thermal regulation of clothes in the future, functional clothing should take thermal sensitivity and sweat rate of different body areas into account and functional fabric, clothing structure, and fabric quality. Moreover, we have done some experiments to explore how much sweat was actually caused by wearing FCPE. Five participants were recruited, and they all completed two trials with 2 clothing ensembles (i.e., combat uniform and FCPE). In all trials, participants exercised on the treadmill (speed 4.0 km/h, incline 5%) in the climatic chamber (35 °C, 60% relative humidity). In combat uniform trials, participants exercised 1 h. In FCPE trials, the trial was stopped when reaching the criteria mentioned in the paper. Results showed that compared with combat uniforms, the rate of sweat evaporation wearing FCPE increased by 92.0%.

**Table 3:**
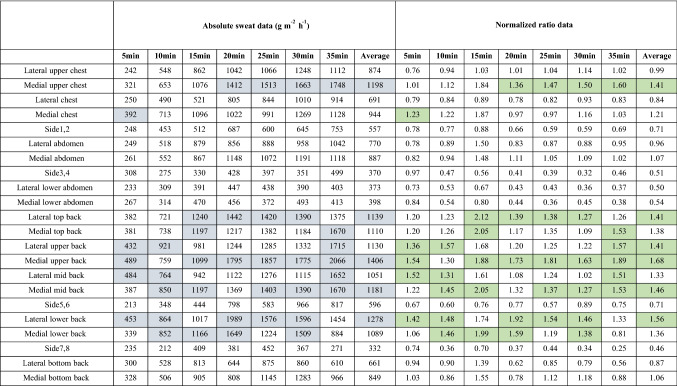
Descriptive statistics for 35 regions sampled.

The top five absolute values are showed by blue shading, and the top five relative values are showed by green shading.

## Conclusions

The primary findings were: (1) wearing FCPE can cause excessive perspiration, and the average weight reduction was 644.5 g as sweating in the upper body rose steadily from the first to fourh period; (2) regions can be categorized into two types based on RSR alteration during exercise. The first includes medial chest, medial abdomen and medial back, evenly sweated throughout the exercise. The second group contains the remaining portions of the body that sweated more during activity, with sweat rates increasing considerably between the first and fourth period of the experiment and sweat rates increased significantly between the fourth and seventh minutes. Furthermore, normalized sweating ratio data showed no obvious changes in the distribution of regional sweat rates to the upper body; (3) when comparing regions, the posterior was found to have a higher sweat rate than the anterior, as well as higher sweat rates on the medial upper chest and back, and lower sweat rates on the waist and bottom.

We have no prior discussions with a *Scientific Reports* Editorial Board Member about the work.

## Data Availability

The data for this manuscript can be obtained from the author upon reasonable request.
